# Long noncoding RNAs: p53’s secret weapon in the fight against cancer?

**DOI:** 10.1371/journal.pbio.3000143

**Published:** 2019-02-13

**Authors:** Emily Dangelmaier, Sarah B. Lazar, Ashish Lal

**Affiliations:** Regulatory RNAs and Cancer Section, Genetics Branch, Center for Cancer Research, National Cancer Institute, National Institutes of Health, Bethesda, Maryland, United States of America

## Abstract

p53 regulates the expression of hundreds of genes. Recent surprising observations indicate that no single protein-coding gene controls the tumor suppressor effects of p53. This raises the possibility that a subset of these genes, regulated by a p53-induced long noncoding RNA (lncRNA), could control p53’s tumor suppressor function. We propose molecular mechanisms through which lncRNAs could regulate this subset of genes and hypothesize an exciting, direct role of lncRNAs in p53’s genome stability maintenance function. Exploring these mechanisms could reveal lncRNAs as indispensable mediators of p53 and lay the foundation for understanding how other transcription factors could act via lncRNAs.

## Abstract 2

Transcription factors regulate hundreds of genes, a subset of which could mediate its effects in a given context. This Unsolved Mystery article explores mechanisms by which long noncoding RNAs might regulate such a subset downstream of p53, a well-studied transcription factor and major tumor suppressor.

## Introduction

Regulation of gene expression plays a crucial role during development and disease. In a given cell, this regulation generally occurs at the level of transcription and is controlled by transcription factors that activate the expression of hundreds of genes to control multiple pathways and diverse phenotypes. In a complex disease such as cancer, is the biology of a transcription factor controlled via a single target gene, controlled via a subset of target genes, or distributed among all its targets? We propose a model according to which a transcription factor mediates its effects via a subset of its target genes. This subset is potentially selected by specific long noncoding RNAs (lncRNAs), an emerging class of regulatory RNAs greater than 200 nucleotides long [1]. This model and the underlying mechanistic scenarios that we propose are based on our current understanding of lncRNAs and recent observations on p53 [2–5], the most well-studied transcription factor and a major tumor suppressor mutated in more than half of human cancers [6–8]. Exploring this model will enhance our understanding of the molecular mechanisms by which gene expression is controlled and lead to the development of improved cancer therapies. In addition to lncRNAs, p53-mediated tumor suppression could also be regulated by p53 binding at enhancers and p53-regulated enhancer RNAs [9–12]. Here, we will focus on the role of lncRNAs in mediating the tumor suppressor effects of p53.

### A lncRNA can regulate a subset of p53 targets. Why?

**Fig 1:**
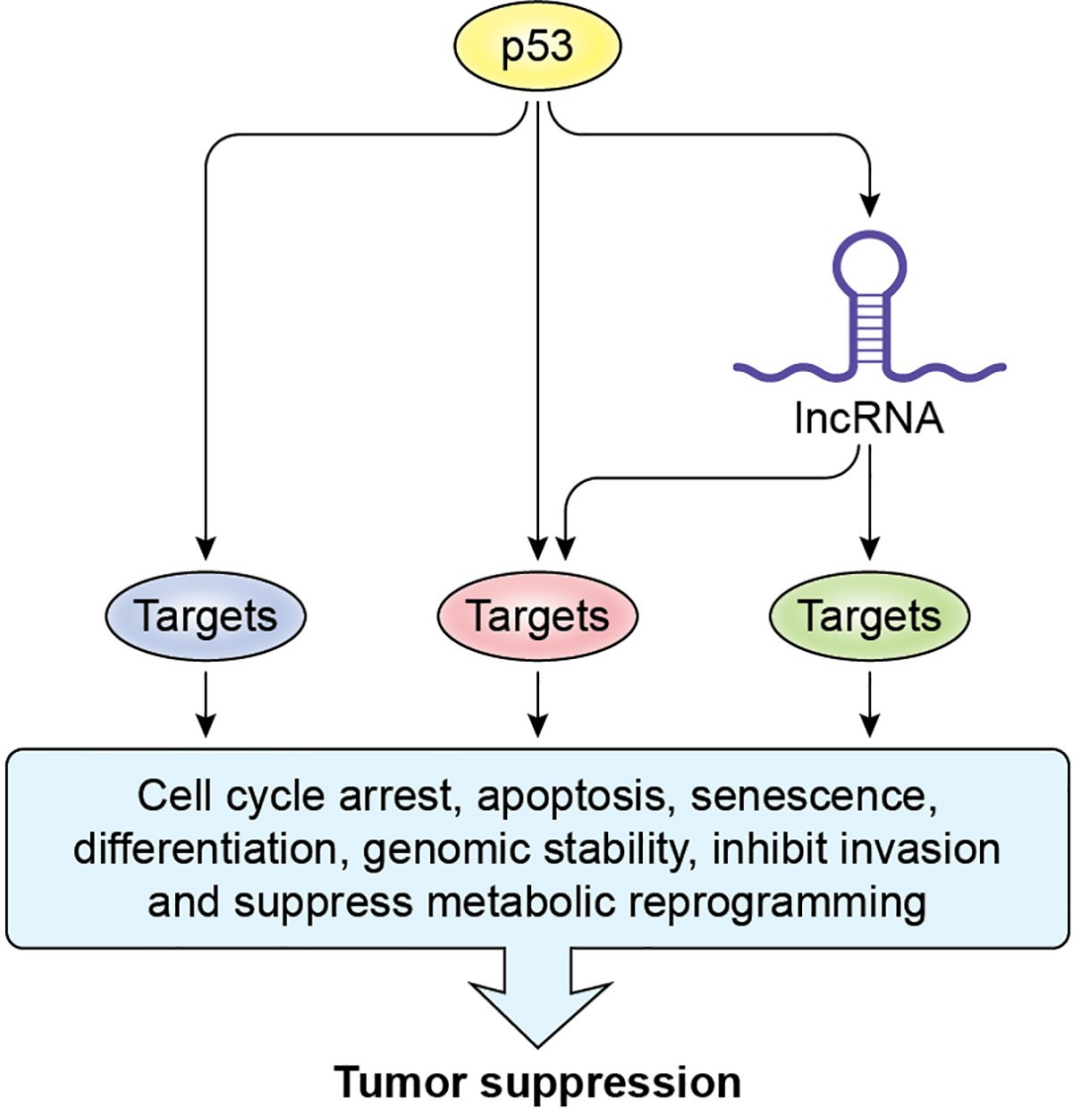
A p53-induced lncRNA can regulate a subset of genes in the p53-regulated transcriptome to play a major role in tumor suppression. lncRNA, long noncoding RNA.

Recent unexpected findings have led to a new search for mechanisms responsible for p53-mediated tumor suppression. p53 has been studied for almost four decades and is known to directly bind to DNA in a sequence-specific manner to induce the expression of a myriad of genes. The outcomes of p53 activation are diverse and include cell cycle arrest, apoptosis, and senescence [13]. Although the genes that mediate these phenotypes downstream of p53 have been identified, recent puzzling observations strongly suggest that they are not sufficient on their own, for p53-mediated tumor suppression in vivo [4, 14–16]. For example, unlike p53-knockout mice that develop highly penetrant spontaneous tumors within six months of age, the triple knockout for *p21*, *Puma*, and *Noxa*—the critical mediators of p53-induced apoptosis, cell cycle arrest, and senescence—are not prone to spontaneous tumor development [5]. Perhaps what is even more surprising are the findings from two recent innovative functional genomics studies in which the authors found that no single protein-coding gene controls the antiproliferative effects of p53 [2, 3]. These studies utilized RNA interference (RNAi) to knock down protein-coding genes, but noncoding RNAs were not targeted. Because some lncRNAs have been shown to directly regulate the transcription of many genes [17, 18] or even an entire chromosome [19, 20], it raises the exciting possibility that lncRNAs play a major role in mediating the effects of p53 by regulating a subset of p53 targets. We propose that some p53-induced lncRNAs control the expression of a subset of genes directly or indirectly up-regulated by p53 and, consequently, p53-mediated tumor suppression (Fig 1). This regulation could be mediated by lncRNAs directly regulated by p53 and/or lncRNAs that are activated in coordination with p53 but not in a direct manner [12].

Why lncRNAs? First, although the majority of lncRNAs transcribed from the human genome have not been studied at the molecular level, some lncRNAs play a crucial role in regulating key cellular processes, including, but not limited to, cellular proliferation, metastasis, differentiation, and genomic instability [21]. Because these processes are also controlled by p53, many lncRNAs have the potential to be functionally integrated into the p53 pathway. For example, we have recently shown that the p53-induced lncRNAs *PURPL* [22] and *PINCR* [23] regulate p53 itself or the induction of a subset of p53 target genes, including *BTG2*, *RRM2B*, and *GPX1*, which are implicated in cell cycle arrest and apoptosis during DNA damage. In addition, the p53-induced lncRNAs *NEAT1* and *PINT* have been recently shown to play crucial roles in p53-mediated tumor suppression [16, 24–28]. Second, because lncRNAs are typically expressed at low levels, it may be that they do not directly regulate the expression of hundreds of genes. Although this argument is very difficult to address experimentally, lincRNA-p21, a well-studied p53-induced lncRNA [29–31], is expressed at eight molecules per cell and has been proposed to lack genome-wide regulatory functions [31, 32]. Therefore, lncRNAs are more likely to regulate a subset of genes within a pathway. Third, just as p53 directly or indirectly controls the expression of genes at various stages transcriptionally and post-transcriptionally, lncRNAs have been shown to modulate gene expression at a variety of stages, often depending on their patterns of subcellular localization. Therefore, lncRNA low abundance and high functional correlation with p53 suggest that lncRNAs likely play a critical role in the p53 pathway by regulating a subset of p53 targets, which control tumor suppressor activities.

### A lncRNA can regulate a subset of p53 targets by associating with DNA. Mechanisms?

**Fig 2:**
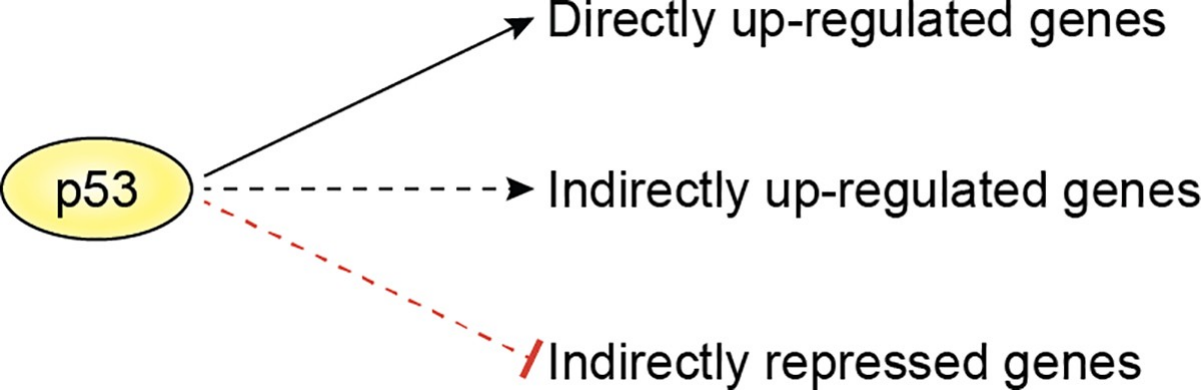
The subset of genes in the p53-regulated transcriptome that a p53-induced lncRNA could regulate. lncRNA, long noncoding RNA.

The p53-regulated transcriptome consists of genes that are directly up-regulated, indirectly up-regulated, and indirectly repressed (Fig 2). The subset of genes in the p53-regulated transcriptome that mediate the tumor suppressor effects of p53 could, in principle, belong to either or all of these categories. A lncRNA could act as an activator and/or repressor of gene expression and modulate the expression of a subset of the p53-regulated transcriptome. What are the molecular mechanisms by which a lncRNA regulates these genes? Perhaps the most widely conceived function of lncRNAs is their association with chromatin to activate or repress gene expression. Therefore, the efficacy of lncRNA function lies in its specificity for target gene loci and must involve mechanisms through which it can associate with chromatin at the intended loci.

**Fig 3:**
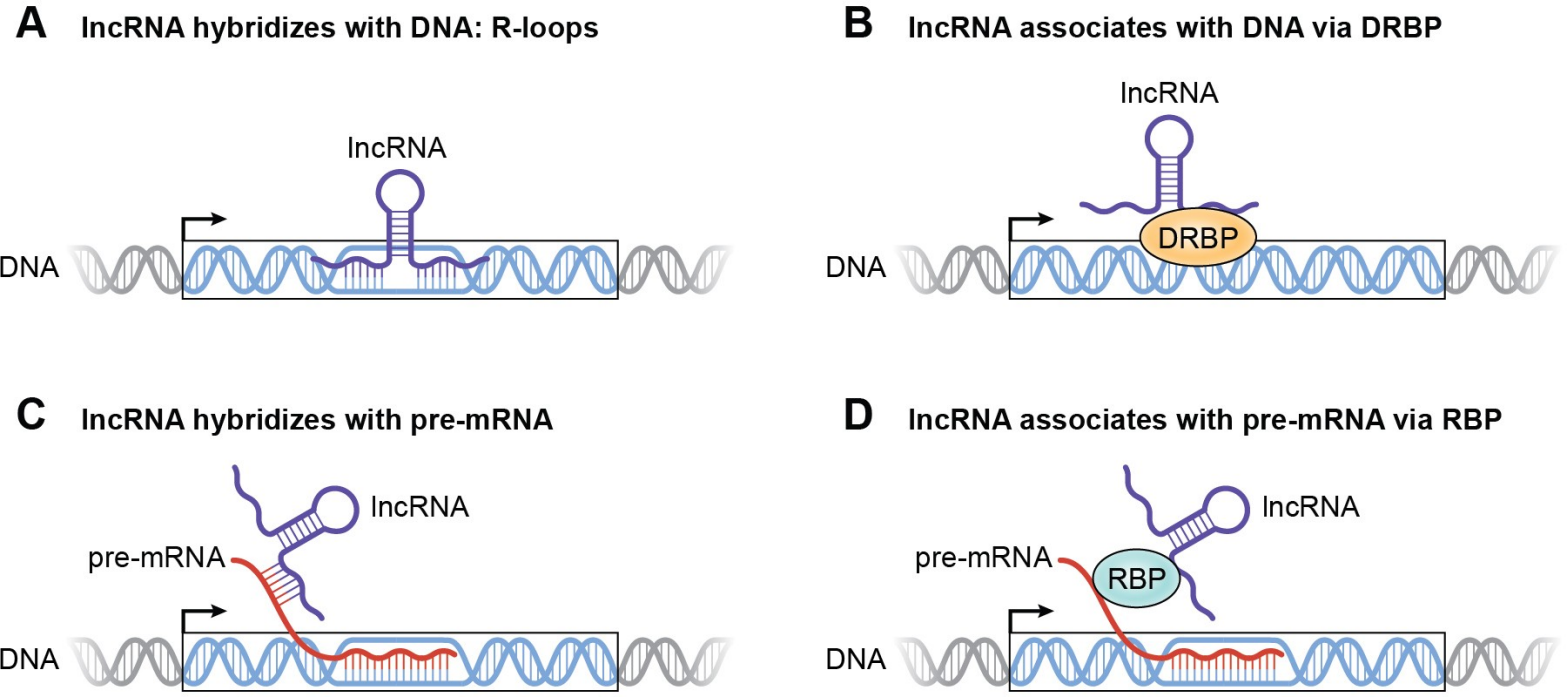
Mechanisms by which a p53-induced lncRNA could modulate gene expression by associating with chromatin at regulatory regions. (A) lncRNA directly associates with DNA by forming R-loops. (B) lncRNA indirectly associates with DNA via a DRBP. (C) lncRNA indirectly associates with DNA by binding to pre-mRNA. (D) lncRNA associates with DNA indirectly by binding to pre-mRNA via an RBP. DRBP, DNA- and RNA-binding protein; lncRNA, long noncoding RNA; R-loop, RNA-DNA hybrid; RBP, RNA-binding protein.

One mechanism through which this could be achieved is via direct association of the lncRNA with chromatin through the formation of RNA-DNA hybrids (R-loops) at regulatory DNA sequences of a subset of p53 targets (Fig 3A). R-loops are thermodynamically favorable and thus represent a plausible mechanism to facilitate direct, sequence-specific binding of the lncRNA at target gene loci [33]. These interactions could occur at regulatory regions of a subset of p53 targets that are hot spots for R-loop formation, including promoters, 1 to 2 kb downstream of the transcription start site or near the polyadenylation sequence at the 3ʹ end of the target gene. Formation of such R-loops by lncRNAs may be aided by sequence elements that promote R-loop formation, such as G-rich sequences in the lncRNA and negatively supercoiled DNA. Alternatively, RNA helicases bound to a lncRNA could facilitate the conversion of intramolecular Guanine-Cytosine (GC)-rich sequences in the lncRNA to single-stranded regions thereby increasing the affinity of the lncRNA for the target DNA. In addition, a lncRNA can be recruited to chromatin by its interaction with DNA- and RNA-binding proteins (DRBPs), a class of proteins that can bind to DNA as well as RNA [34]. In this case, the DNA sequence recognized by the lncRNA will be determined by the specific binding motifs in the DRBP (Fig 3B).

Through these mechanisms of lncRNA association with chromatin, a lncRNA will be able to interact with regulatory regions of p53 targets in a sequence-specific manner. Where could these regulatory regions be in the genome? These regions could be in enhancers, promoters, insulators, or the gene body of a subset of p53 targets. In this way, a lncRNA may recruit transcriptional regulators to aid in transcription, thus increasing the expression of certain p53 target genes. A lncRNA may also increase target gene expression by facilitating chromatin looping. In this case, the lncRNA can interact with enhancer regions to increase the transcriptional activity of p53 targets by altering chromatin structure. For example, *PINCR* likely plays a role in the formation of chromatin loops between the promoters and enhancers of the p53 targets *BTG2*, *GPX1*, and *RRM2B* [23]. Additionally, lncRNAs may influence histone modifications at regulatory sites to repress or induce gene expression. This regulation can be achieved through lncRNA interactions with chromatin-modifying enzymes. lncRNAs could also facilitate association of RNA-binding proteins (RBPs) with chromatin to regulate the expression of a subset of p53 targets. For example, the lncRNA *XIST* has been shown to bind to several RBPs to achieve X-chromosome inactivation [35].

### A lncRNA can regulate a subset of p53 targets by associating with pre-mRNAs. How?

A lncRNA can indirectly associate with chromatin via interactions with pre-mRNAs of a subset of p53 targets. RNA–RNA interactions are extremely stable, indicating that this can be a significant mechanism through which lncRNAs associate with chromatin. These interactions may be direct, involving sequence-specific binding of the lncRNA to the pre-mRNA (Fig 3C). Alternatively, this pre-mRNA–facilitated association of the lncRNA with chromatin could occur indirectly via RBPs that recognize specific motifs in the pre-mRNA and the lncRNA (Fig 3D). For example, *MALAT1*, a nuclear-speckle–localized lncRNA, has been shown to interact indirectly with pre-mRNAs through RBPs [36]. These direct or indirect RNA–RNA interactions will allow for sequence-specific control of a subset of p53 target genes at the post-transcriptional level, resulting in facilitating or inhibiting mRNA splicing and/or processing by a p53-induced lncRNA.

### A lncRNA could regulate a subset of p53 targets beyond interactions with chromatin. How?

The mechanisms of associating with chromatin outlined above may represent the most commonly utilized mode by which nuclear-retained lncRNAs dictate a subset of p53 targets. However, lncRNAs that localize to the cytoplasm also have the potential to exert similar functions. Some cytoplasmic lncRNAs are known to promote either mRNA stability or degradation [37]. A p53-induced lncRNA could bind sites targeted by the mRNA degradation machinery to increase the stability of mRNAs in the p53-regulated transcriptome. Alternatively, the lncRNA may recruit degradation machinery and bind to such mRNAs to decrease stability. These lncRNA–mRNA interactions could also occur indirectly via RBPs that provide sequence specificity. For example, mRNAs of specific p53 targets may be destabilized by lncRNAs under normal conditions but stabilized by lncRNAs in response to p53 activation. Additionally, lncRNAs could promote or inhibit the translation of a subset of p53-regulated mRNAs on polysomes. For example, the human lncRNA RoR has been shown to repress p53 mRNA translation [38]. Finally, a lncRNA that localizes to the cytoplasm may alter mRNA levels by acting as competing endogenous RNA (ceRNA) [39]. In this scenario, the lncRNA may prevent interaction of a microRNA (miRNA) with its target mRNAs by outcompeting mRNAs for shared miRNAs.

An important point to consider with each of these mechanistic scenarios is the copy number per cell of the lncRNA that is being considered. The p53-induced lncRNA and the target miRNA or protein should be present in stoichiometric amounts to substantially affect the expression of the target gene. Perhaps the best example of a cytoplasmic lncRNA in which the issue of stoichiometry was carefully considered is *NORAD*, a very abundant lncRNA that sequesters Pumilio proteins to maintain genome stability [40].

### Direct regulation of DNA repair by a p53-induced lncRNA. How?

One of the hallmarks of cancer is DNA double-strand breaks (DSBs), which, if not repaired accurately, lead to mutations and chromosomal rearrangements [41]. DSBs are generally repaired by homologous recombination (HR) or nonhomologous end joining (NHEJ). The preferred substrate for HR is the sister chromatid, but this substrate is only available during the S and G2 phases of the cell cycle. Therefore, during G0/G1, NHEJ can be used to join broken DNA ends without the use of a template DNA, but this is error prone. Alternatively, to repair DNA by HR in G0/G1 phase of the cell cycle, a cell can potentially use endogenous RNA as a substrate for DNA synthesis. Although direct involvement of RNA in DNA repair was unanticipated and considered a rare mechanism, there is now mounting evidence that endogenous RNAs can serve as a template for DSB repair [42–47]. We propose that, in addition to the known roles of some lncRNAs—such as *DDSR1* and damage-induced lncRNAs (dilncRNAs) in DNA repair pathways [48, 49], and *NORAD* in the maintenance of genome stability [40, 50]—specific antisense lncRNAs play direct roles in DSB repair by base-pairing to the damaged DNA.

RNA-mediated DNA repair reverses the central dogma in which DNA is synthesized by RNA. These repair mechanisms, therefore, could rely on RNA interacting with a reverse transcriptase (RT). Although this interaction is well established in the context of retroviruses, retrotransposons, and during telomere synthesis, reverse transcription is likely not limited to these scenarios. Nearly half of the noncoding human genome consists of repetitive elements [51], and over 75% of lncRNA sequences contain elements derived from retrotransposons [52]. Therefore, the majority of lncRNAs contain sequences that are known to interact with RT in the context of DSB repair. One could imagine that genes that play more significant roles in cellular pathways may contain antisense lncRNAs to preserve their integrity. Taken together, this evidence leads to the hypothesis that lncRNAs play crucial roles in “guarding the genome” by functioning in DSB repair pathways.

p53 is known to play a crucial role in maintaining genomic stability [53]. A recent report that utilized in vivo RNAi screening on select p53 targets provided strong evidence that in some contexts the genome stability function of p53 is regulated by the DNA repair gene Mlh1, and the regulation of the DNA repair process plays a very important role in p53-mediated tumor suppression [54]. Most lncRNAs were not included in the RNAi library. This raises the following question. In addition to the regulation of the DNA repair function of p53 via Mlh1, can a p53-induced lncRNA also play a direct role in DSB repair? How could this occur, and what could be the advantages over other mechanisms? p53 is the guardian of the genome [13, 55, 56]. It accomplishes its genome stability maintenance function by a variety of well-established mechanisms, including direct transcriptional regulation of specific genes that control cell cycle arrest, apoptosis, senescence, and DNA repair. For example, upon DNA damage, induction of cell cycle arrest by p53 ensures that DNA replication is turned off, thereby allowing more time for the damaged DNA to be repaired. If the DNA damage is too severe to be repaired, p53 kills the cell by inducing apoptosis. In cancer cells that express dysfunctional p53 or have no p53, these processes are disrupted leading to increased genome instability.

For the regulation of these processes via a protein-coding gene, the p53 target gene(s) would have to be transcriptionally induced by p53, and the corresponding mRNA would then be exported to the cytoplasm and translated. The effector protein may need to be post-translationally modified and, in some cases, imported to the nucleus. We propose that a p53-induced lncRNA could provide a faster and more efficient mechanism of fixing the damaged DNA by directly facilitating lncRNA-mediated DNA repair in the nucleus.

**Fig 4:**
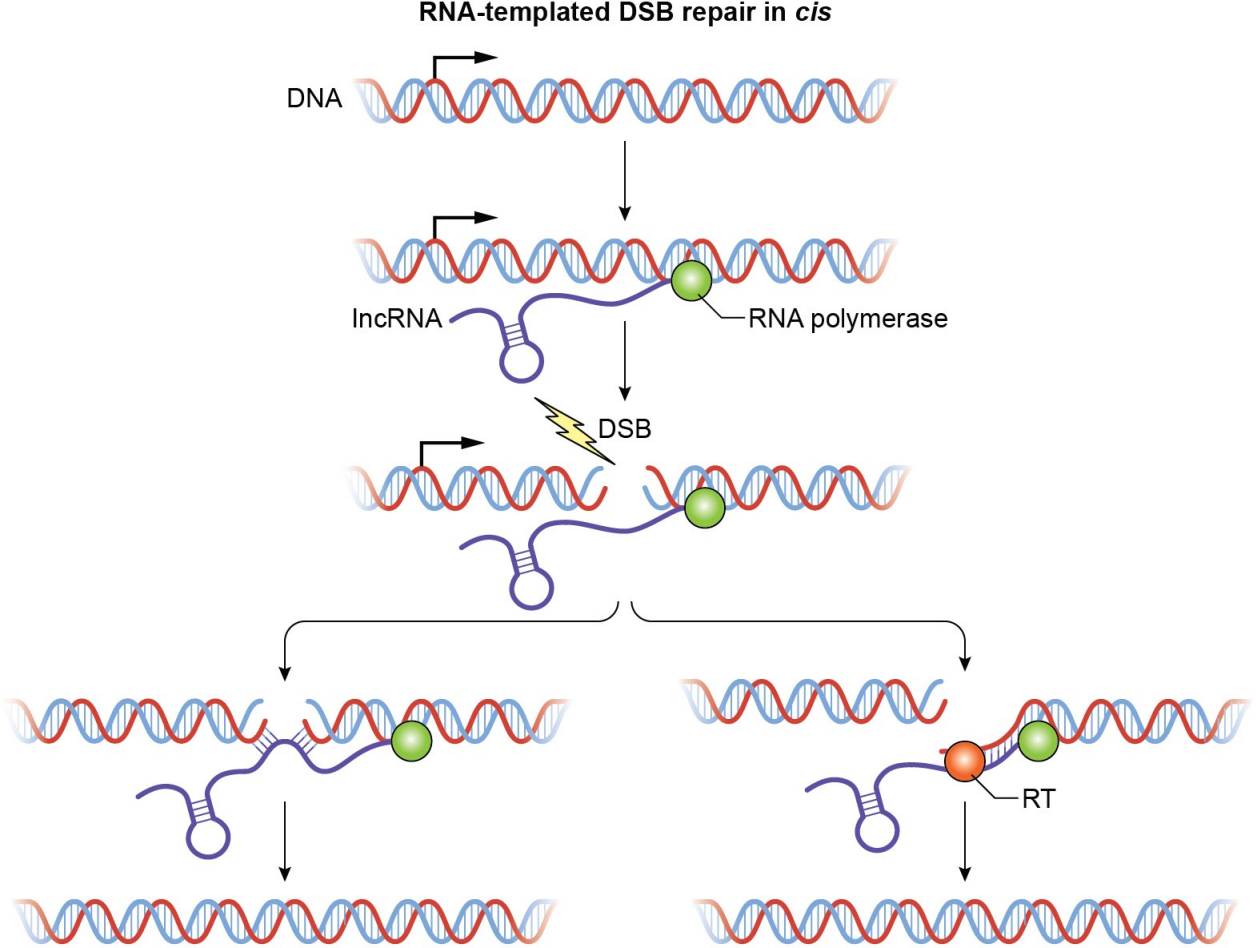
Regulation of DSB repair in *cis* by a p53-induced antisense lncRNA. The antisense lncRNA binds DNA at either side of the break and facilitates end joining or acts as a template for resynthesis of damaged DNA. The RNA polymerase that transcribes the antisense lncRNA is shown as a green circle; the RT for synthesis of cDNA using the lncRNA as template is shown as an orange circle. DSB, double-strand break; lncRNA, long noncoding RNA; RT, reverse transcriptase.

How could this occur? The repair may be templated or nontemplated and will utilize a lncRNA transcript homologous to the DNA flanking the DSB. This transcript could be an antisense lncRNA transcribed from the damaged locus prior to DNA damage or from the undamaged homologous allele in response to DNA damage. An exciting possibility is that the antisense lncRNA could pair with homologous DNA at the site of a DSB. In *cis*, a lncRNA could base-pair with both sides of the DSB and facilitate end joining (Fig 4). This lncRNA–DNA interaction at the site of the break would occur by forming R-loops. HR machinery has been shown to modulate R-loop formation in the context of genomic instability [57]. Additionally, RNA–DNA hybrids have been shown to promote HR [58]. In *trans*, an antisense lncRNA or a lncRNA that shows partial complementarity to the regions near the damaged DNA may associate with chromatin at the site of the DSB through a DRBP or directly by forming R-loops. Although both scenarios are possible, lncRNA repair in *cis* would be preferred because a transcript from the same site as the DSB would have greater repair frequency.

In addition to facilitating end joining, an antisense lncRNA could also act as a template for DNA repair, allowing for extension of one of the free DNA ends using a currently unidentified RT (Fig 4). A major advantage of lncRNA-mediated DSB repair could be that only a single molecule of the antisense lncRNA will be required. Our proposed models of lncRNA-mediated DNA repair could be exciting, prevalent, and largely unexplored mechanisms that may unmask the functions of hundreds of currently functionally uncharacterized lncRNAs.

### Conclusions and beyond

We propose that a subset of p53 targets regulated by a lncRNA are required for effective tumor suppression. This hypothesis is supported by the low abundance of lncRNAs and their functional correlation with p53. As described above, lncRNAs may control this subset of p53 targets through direct association with chromatin or indirect association with chromatin via nascent pre-mRNA; these interactions may be facilitated by RBPs or DRBPs. Moreover, lncRNAs could modulate p53 targets at the level of translation via interactions with miRNAs or mRNAs on polysomes. Through these mechanisms, lncRNAs may be necessary for effective p53 tumor suppression through regulation of the expression of a subset of p53 targets. Our proposed model on a potential role of antisense p53-regulated lncRNAs in DNA DSB repair, in addition to the known role of Mlh1 in DNA repair downstream of p53 [54], is exciting to explore given the fact that p53 is the guardian of the genome. It is likely that these roles of lncRNAs may be broadly applicable to other transcription factors, accounting for the wide array of lncRNA cellular functions and a significant number of lncRNA genes in the human genome. Therefore, lncRNAs may represent integral regulators of transcription factor pathways and may be necessary for carrying out the observed functions of such transcription factors to play critical roles in development and disease.

## Abbreviations

ceRNA: competing endogenous RNA
dilncRNA: damaged-induced lncRNA
DRBP: DNA- and RNA-binding protein
DSB: double-strand break
GC: Guanine-Cytosine
HR: homologous recombination
lncRNA: long noncoding RNA
miRNA: microRNA
NHEJ: nonhomologous end joining
RBP: RNA-binding protein
R-loop: RNA-DNA hybrid
RNAi: RNA interference
RT: reverse transcriptase
